# Sexuality education for young people in Germany. Results of the ‘Youth Sexuality’ representative repeat survey

**DOI:** 10.25646/9875

**Published:** 2022-06-29

**Authors:** Sara Scharmanski, Angelika Hessling

**Affiliations:** Federal Centre for Health Education, Department S – Sexuality Education, Contraception and Family Planning, Unit S3 – Task Coordination, National and International Cooperation, Research and Training, Cologne, Germany

**Keywords:** ADOLESCENTS, PREVENTION, CONTRACEPTION, REPRODUCTIVE HEALTH, SEXUAL EDUCATION

## Abstract

The Federal Centre for Health Education (BZgA) has been conducting the ‘Youth Sexuality’ representative survey on a regular basis since 1980. This continuous monitoring generates insights into the sexual and reproductive health of young people in Germany and constitutes an important basis for evidence-based health communication.

A total of N=6,032 young people between the ages of 14 and 25 participated in a combination of oral and written interviews (Computer Assisted Personal Interviews (CAPI)).

As primary sources of knowledge for, adolescents state that they obtain information through school lessons (69%), personal discussions (68%), and the Internet (59%). In addition to these sources, professional gynaecological counselling and sexuality education at home are also important sources of information. To what extent trusted contact persons are available in the family depends heavily on the adolescents’ sociocultural backgrounds.

Providing information and disseminating knowledge to young people in the field of sexual and reproductive health is organised intersectorally in Germany. In this way, it is possible to also reach those who do not have any contact persons at their disposal in their direct family. Maintaining and strengthening the current commitment in promoting sexual health is of key importance, as only this will ensure the next generation’s sexual and reproductive health, and provide an evidence-based counterbalance to anecdotal information, especially in the digital domain.

## 1. Introduction

Promoting and ensuring sexual and reproductive health is one of the key goals of the Sustainable Development Goals of the World Health Organization (WHO) [1]. The Declaration of 2015 explicitly includes the access to contraception counselling as well as to family planning and sexuality education information. Since 1992, the Federal Centre for Health Education (BZgA) has been commissioned under the Pregnancy Conflicts Act (SchKG) to develop concepts for sexuality education and provide free information relating to contraception nationwide [2]. These materials for sexuality education reach the target groups either directly or are deployed by disseminators within the framework of sexuality education offerings.

Within the BZgA, conducting and promoting large representative studies to evaluate and align the measures for sexuality education have a long tradition [3–6]. In this context, the representative cross-section survey relating to youth sexuality, which has been conducted on a regular basis since 1980, is an important monitoring tool [7]. Based on this survey data, information can be obtained about current sexual and contraception behaviours, as well as aspects of sexuality education. After all, only when evidence-based findings are available, can target group-specific needs be identified, discussions be directed in a target group-specific manner, the effectiveness be verified, and the necessary strategic and operational realignment be made.

Especially ‘first-time sex’ is a heavily discussed topic in society. The data from the 9th iteration of the Youth Sexuality Study from 2019 clearly shows that the percentage of adolescents with (heterosexual) sexual intercourse experiences has not changed in the last decades. In fact, in the age groups of 15- and 16-year-olds, it has declined significantly [8]. This once again confirms the trend that young people are more sexually restrained than ten years ago [7, 9] and almost all adolescents in Germany use contraception: In 2019, only 9% indicated not having used contraception during their very first sexual intercourse, and 5% during their most recent sexual intercourse [8]. Compared to the average of 30 European and non-European industrialized nations, this percentage is very low indeed [10].

The choice of the contraceptive is related to the age and the associated level of sexual experience or the existence of a partnership, respectively. At a young age and with little sexual experience, adolescents especially use condoms as a contraceptive, more rarely the contraceptive pill. With increasing age and the existence of a longer-lasting partner relationship, the frequency of pill use increases significantly [8, 11]. However, even though many young people often use the pill as a contraceptive, especially in partnerships, current data points to a possible change in mindset about hormonal contraception: The percentage of respondents using the pill for contraception is declining [8]. This development is consistent with the decline of prescriptions for the pill among girls and young women insured under the statutory health insurance [12]. However, it is not only the frequency of pill use that is declining, its health and safety is rated more negatively [8], and health-related aspects play a relevant role with regard to a conscious lifestyle [13] as well as in the selection of the contraceptive method [14].

But where do young people in Germany currently obtain their knowledge about sexuality and contraception? Which persons, institutions and media contribute to knowledge building, and which role does the Internet play in this context? These and other questions will be answered in this article based on the data from the 9th iteration of the Youth Sexuality Study by the BZgA.

## 2. Methodology

### 2.1 Sample design and study conduct

The present cross-section survey relating to youth sexuality has been repeated regularly for nearly 40 years, whereby the basic methodological framework remained largely unchanged. The data collection of the present 9th iteration was conducted between May and October 2019 by Kantar GmbH using the CAPI (Computer Assisted Personal Interviewing) methodology for combined oral-written interviews. The standard questionnaire was completed in a personal face-to-face interview, while the adolescents and young adults completed more intimate questions using a laptop (self-completed part).

The survey took place in the home environment of the adolescents or young adults, respectively, and mostly without the presence of a third person. In the case of minors, the parents were present at home during the interview. This ensured that when the adolescents wanted more in-depth information about sexuality and contraception following the interview, contact persons were theoretically available to them.

The guardians as well as the adolescents or young adults were informed comprehensively verbally and in writing in advance about the object and purpose of the study. The interview was voluntary and only took place after consent by the parents and the adolescents or young adults. The data acquisition and processing took place in accordance with the currently valid provisions of the General Data Protection Regulation (DSGVO). Personal data, which was deleted permanently from all data carriers immediately after conclusion of the field phase, was acquired and processed only to control field access.

Intensive training prior to conducting the interviews, as well as the many years of experience of the field institute’s staff members in this area of research ensured that the interviewers were able to conduct the interview in an age-appropriate, culturally-sensitive, and empathetic manner.

According to the sample design, eight disproportional partial samples, each resulting from the combination of the three main criteria, being sex (female vs. male), age group (14- to 17-year-olds vs. 18- to 25-year-olds) and cultural origin (with vs. without migration background), were realised in the present 9th iteration of the Youth Sexuality Study. N=2,024 girls and N=1,532 boys between the ages of 14 and 17 as well as N=1,580 young women and N=896 young men between the ages of 18 and 25 participated in the survey. Due to the method of the Youth Sexuality Study, a further non-binary gender differentiation had to be refrained from. The authors would like to stress here that this approach is solely the result of methodological necessities and not of a lack of a diversity-sensitive perspective.

The respondents’ level of education was operationalised through the attended school and/or the highest aspired or obtained level of education, respectively. A migration background was assumed when adolescents or young adults themselves or at least one parent were born without having German citizenship [15].

The selection of the target subjects took place in a non-randomised manner according to the quota method [16], whereby the quota were taken from different Census Bureau publications (cut-off date: December 31, 2017) [17–19]. The parameters of the quota method are sex, age, area of residence, cultural origin, and level of education or type of attended school/obtained highest level of education.

The geographic location of the interviewers was used to ensure an adequate regional distribution, whereby the criteria of federal state, administrative district and city size were correlated relative to the master sample of the ‘Arbeitskreis Deutscher Markt- und Sozialforschungsinstitute e.V.’

### 2.2 Statistical methodologies

To prepare the data sets for the statistical analyses, it was necessary to transfer the disproportional sample design into a proportional one with the help of design weighting factors. Census Bureau publications were used as the basis for the determination of the weighting factor here as well [17–19]. Combined regional, sex, and education weighting factors were applied to the data set. In addition, weighting factors according to nationality group were applied to the group of respondents with a migration background. The design weighting factors range from 0.39 to 2.72. All results published in this article are reported with this design weighting.

Descriptive analyses provide information about features of sexuality education and contraception counselling of adolescents and young adults in Germany. The questions of the used items can be found in the Annex Table 1. In addition, two-sided χ^2^ tests were used to analyse the significance of different distributions in subgroups or between individual trends, respectively. In some cases, differentiation was applied according to sociodemographic features (especially religious denomination, religious bond and highest obtained or aspired education level), if statistically relevant differences were at hand. Statistical significance was assumed starting at an **α** error level of less than 5% (p<0.05). Statistical analyses were conducted using IBM SPSS, version 25.

In case long-term trends were represented, the subsample of the adolescents between the ages of 14 and 17 without a migration background was used, because trend data from almost 40 years was available for this subsample.

## 3. Results

### Sources of sexuality education

**Figure 1:**
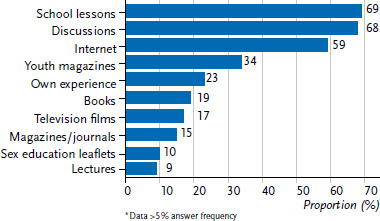
Sources of sexuality education (N=3,556 14- to 17-year-olds, unweighted)^*^ Source: Youth Sexuality Study, 9th iteration (BZgA)

Current data from the Youth Sexuality Study show that the most important sources for sexuality education for adolescents between the ages of 14 and 17 are school lessons, personal discussions and the Internet (Figure 1). The data does not differ significantly between the sexes.

Sexuality education at school and through the Internet will be examined in more detail in the following paragraphs. Furthermore, communication about sexuality is brought into focus. Discussions and counselling at home, at certified counselling centres and in gynaecological counselling will be examined.

### Sexuality education at school

According to information they provided, the adolescent respondents have predominantly obtained their knowledge about sexuality, reproduction and contraception during school classes (Figure 1). A total of 87% of girls and boys between the ages of 14 and 17 currently indicate having discussed sexuality education topics in class. Therefore, school is capable of reaching the vast majority of adolescents with sexuality education content. Compared to the last survey five years ago, however, this constitutes a decline. With 93% each, significantly more girls and boys still reported suitable classroom content in 2014.

**Figure 2:**
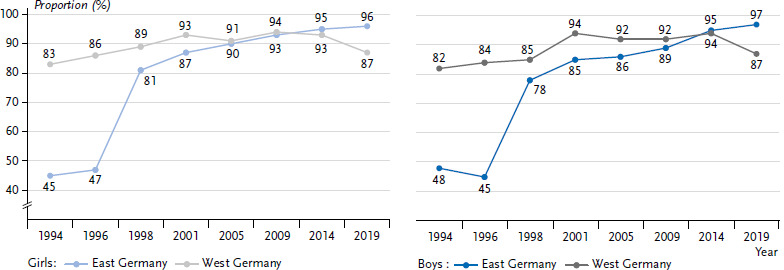
Sexuality education lessons by trend and differentiated by region (14- to 17-year-olds with German citizenship, as of 2014 without a migration background) Source: Youth Sexuality Study, 9th iteration (BZgA)

This trend does not apply to all regions of Germany equally. While in the eastern states 96% of all girls and boys currently state that they have had sexuality education lessons, this is only 86% in the other states (Figure 2). In 2019, the percentages of adolescents indicating that they had had sexuality education lessons at school, therefore differed significantly between the western and the eastern states.

**Figure 3:**
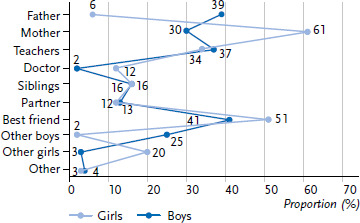
Persons for sexuality education (N=3,556 14- to 17-year-olds, unweighted) Source: Youth Sexuality Study, 9th iteration (BZgA)

In addition to the educational content at school, sexuality education still takes place via communication in the form of personal discussions (Figure 1). For the adolescents between the ages of 14 and 17, their peers (65%) as well as their own parents (56%) are the most important persons in terms of sexuality education (Figure 3), but teachers are also highly relevant in this context. Among girls (34%) as well as boys (37%), they are the third most often mentioned. The significance of teachers in the context of sexuality education has been relatively stable for years. The proportional values for girls have been fluctuating by up to six, and for boys by up to eight percentage points.

On average, however, teachers are considered to be less important for sexuality education by adolescents who have or aspire a low level of education (25% compared to 39% or 35% in the case of a medium or high level of education, respectively). For those girls and boys however, sexuality education at school is particularly important as the parents of adolescents with a lower obtained or aspired level of education are available as a source of sexuality education significantly less frequently: 42% of them mention their own mother or father in this context. By comparison, adolescents with a medium or higher (aspired) level of education name their parents as important persons for sexuality education, 54% and 61%, respectively.

For adolescents with a migration background, teachers are also important contact persons for questions about sexuality and contraception, because for girls and boys with an immigration history, the parents (38%) count as important persons for sexuality education significantly less frequently than for their age peers without a migration background (64%). However, teachers are equally regarded as contact persons, irrespective of migration status. Adolescents with a migration background name them just as frequently (36%) as their age peers without an immigration history (35%).

Both sexuality education at school and teachers serving as contact persons for questions about sexuality and contraception thus play an important role, especially for adolescents who lack contact persons at home more often. Sexuality education at home will be examined in more detail below.

### Sexuality education at home

As Figure 3 shows, parents still play the most important role in sexuality education: 56% of adolescents indicate that their parents are among the most important persons for education about sexual matters.

For girls between the ages of 14 and 17, the mother (61%) is still the most important person to go to for sexuality education. The best friend is most likely also consulted (51%). Beyond that, all other persons play a minor role (Figure 3).

Boys between the ages of 14 and 17 have different preferences: The father (39%) belongs to the inner circle of the most important persons for sexuality education almost as frequently as the best friend (41%). From the boys’ perspective, teachers have a comparatively similar importance (37%) as their fathers.

There is a noticeable trend that today, mothers are less important for their sons as contact partners than 15 years ago, when they were the most important persons to discuss sexuality with (2005: 42%). In the current survey, the percentage of the male respondents mentioning their father as an important contact person, is higher for the first time (39% compared to 30%).

**Figure 4:**
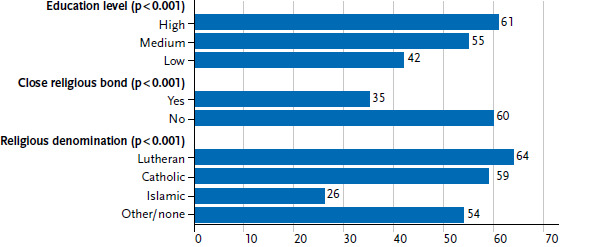
Persons for sexuality education (parents, total share of mother and father) (N=3,556 14- to 17-year-olds, unweighted)^*^ Source: Youth Sexuality Study, 9th iteration (BZgA)

The current data from the Youth Sexuality Study also shows, however, that sexuality education at home is strongly associated with religious-cultural origins as well as the adolescents’ obtained or aspired level of education (Figure 4). Adolescents with low obtained or aspired levels of education, close religious bonds and/or Islamic denomination name their parents significantly less frequently as important contact persons for sexuality education than respondents of other groups.

**Figure 5:**
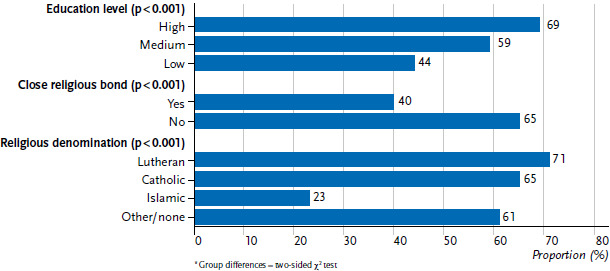
Addressing sexuality with family (N=3,556 14- to 17-year-olds, unweighted)^*^ Source: Youth Sexuality Study, 9th iteration (BZgA)

Significant differences with regard to the adolescents’ religious bonds as well as the obtained or aspired levels of education can also be observed when asked to what extent they are able to discuss sexuality and partnership with family members (Figure 5). In total, more girls than boys say that they can talk about these matters with family members (64% vs. 58%).

In summary, the data from the Youth Sexuality Study show that education about sexuality also still happens at home and in the family environment for many young people, but the extent to which parents are contact persons and the ones providing sexuality education, is strongly associated with sociocultural origin.

In addition to the already mentioned sources for sexuality education, young people also indicate the Internet as a source of information about sexuality and contraception (Figure 1). The Internet as a source of information will be brought into focus below.

### The Internet as a source of sexuality education

The Internet is an important medium for young people to socialise on and obtain information from. The data from the Youth Sexuality Study also confirms this. The significance of the Internet as a source for sexuality education has been increasing gradually since 2001. In 2001, 3% of the girls and 10% of the boys indicated utilising the Internet as a source of sexuality education. By 2019, the percentage had increased to 56% for girls and 60% for boys. In the current iteration Youth Sexuality Study trend, the Internet is thus the third most important source of information about sexuality and contraception for young people in Germany (Figure 1).

The Internet is also where young people want to look for additional information: 66% of the 14- to 17-year-olds and 70% of the young adults between the ages of 18 and 25 indicate this.

The Internet is not only a preferred source of knowledge, but the information that young people find there is in fact also important to them: Two out of three underage adolescents (65%) and almost three out of four young adults (73%) indicate that they have actually already found something on the Internet that was important to them about sexuality.

When young people look on the Internet for information about sexuality and contraception, they use search engines (they ‘google’). 79% of male and 83% of female adolescents and young adults between the ages of 14 and 25 indicate this. Googling outscores all other options. Wikipedia is a site where 16% of girls and young women, 19% of boys and young men start their search for information. One out of seven girls and young women (14%), but at least one-fifth of the boys and young men (22%) use YouTube as their first place to go – even more so among young adult men (24%) than among male minors (19%). In comparison, 15% or 14%, respectively, of girls and young women indicate this. Facebook, Instagram and Twitter, however, are channels that young people use much more rarely as their first place to go in their search for information about sexual topics (in each case below 5%).

**Figure 6:**
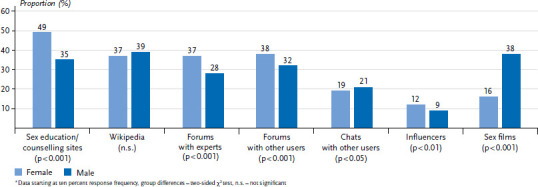
Internet sources used by adolescents and young adults (N=4,112 14- to 25-year-olds, who indicate having found out something important about sexuality on the Internet, unweighted)^*^ Source: Youth Sexuality Study, 9th iteration (BZgA)

The current survey iteration of the Youth Sexuality Study furthermore shows that young men and young women use the Internet’s range of information significantly differently (Figure 6). Female respondents use sexuality education or counselling sites, but also Wikipedia and Internet forums with experts or other users. This is different among boys and young men. They do not have one predominant source of information. For them, sex films and Wikipedia are means to obtain valuable information. The percentages of those who have visited sexuality education or counselling sites or who have shared their ideas in forums with other users or with experts, respectively, are similarly high (Figure 6).

When looking exclusively at the information sourcing behaviour of adolescents under the age of 18, however, a somewhat different picture presents itself. Wikipedia simply as a reference guide for information is just as popular as specific sexuality education and counselling sites (in each case 41%). It turns out, however, that adolescents indicate significantly more frequently than young adults (17% compared to 8%) and girls between the ages of 14 and 17 more frequently than boys of the same age (20% compared to 15%) that they found out something about sexuality that was relevant to them from influencers. It is not the case, however, that especially those adolescents who do not have any contact persons at home or persons of trust otherwise, are guided by influencers. Such a significant connection cannot be recognised in the data (person of trust: ‘available’ 18%, ‘not available’ 14%; Parents as contact persons for sexual questions: ‘yes’ 15%, ‘no’ 19%).

By their own account, sex films are important places to go for knowledge about sexuality, especially for male adolescents. Among 14- to 17-year-olds, 37% of male adolescents indicate that they have found out something important about sexuality when watching sex films. 16% of girls of the same age report this significantly less, while at the ages between 14 and 17, it is especially boys with a low education who name sex films as important sources of information. Almost half of them indicate this (48% compared to 38% among a medium or 32% among a high obtained or aspired level of education).

### Contraception counselling in counselling centres and medical surgeries

Germany has a tight net of counselling centres. Each individual is entitled to obtain complementary information and counselling on matters concerning sexuality education, contraception, family planning and pregnancy in specialised counselling centres. Counselling centres also support other institutions offering sexuality education, such as schools.

The data from the current trend shows that adolescents between the ages of 14 and 17 accept the counselling centres’ expertise. In the Youth Sexuality Study, 19% of girls and 18% of boys indicate that counselling centre experts are among their preferred authorities for sourcing knowledge about sexual matters. Contact persons at counselling centres are particularly important to slamic adolescents and/or adolescents with strict denominational bonds: 20% of Islamic respondents and 21% of adolescents with strict religious bonds name experts in counselling centres as their preferred contact persons to obtain information on sexuality from. This is remarkable to the extent that these adolescents are not able to discuss matters of sexuality and contraception in their family environments as much as their peers in the other groups (see chapter Sexuality education at home). Counselling centres can therefore fill a significant void, especially for adolescents and young adults who lack contact persons in their family environments. This applies to boys to an event higher extent than to girls. After all, in terms of professional contraception counselling, girls can also turn to gynaecologists.

In addition to certified counselling centres, medical specialists play an important role in sexuality education and contraception counselling. Health care professionals – in this context mostly gynaecologists - are relevant contact persons for over 10% of 14- to 17-year-old girls. To boys of the same age this applies only in exceptional cases (2%) (Figure 3).

Parallel to the general trend of having one’s first sexual experience at a later age, the first visit to the gynaecologist now also takes place later than five years ago. In 2014, girls and young women between the ages of 14 and 25 visited a gynaecologist for the first time on average at the age of 13.1. Today, the interviewed girls and young women indicate an average age of 15.0.

In the overall group of 14- to 25-year-old girls and young women, 84% indicate already having visited a gynaecologist. Here, however, there are also differences with regard to the sociocultural background of the respondents. Islamic girls and young women and/or girls and young women with strict religious bonds indicate significantly more frequently that they have visited a gynaecological surgery (Table 1).

**Figure 7:**
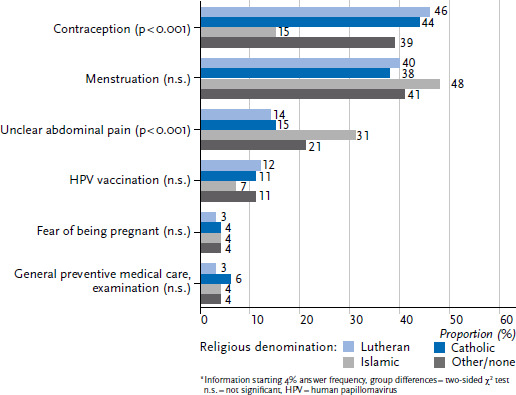
Reason for visiting a gynaecologist for the first time (n=2,797 14- to 25-year-old young women, who visited a gynaecologist, unweighted)^*^ Source: Youth Sexuality Study, 9th iteration (BZgA)

Asked about their motives for visiting a gynaecologist, almost half of the girls and young women between the ages of 14 and 25 name matters of contraception (41%), followed by menstrual problems (40%). Differentially speaking, there are also differences, depending on the cultural-religious socialisation of the respondents (Figure 7).

The data from the Youth Sexuality Study furthermore shows that those who have access to a trusted person, also include the doctor significantly more frequently in their contraception counselling than those who have nobody to talk to about sexual matters (62% compared to 34%). Also, those who can communicate openly about matters of contraception at home are more likely to consider visiting a doctor for contraception counselling (70% compared to 47%).

In summary, this demonstrates that many young people value the expertise of recognized counselling centres, and name it as a preferred source of knowledge when lacking information. This preference is expressed independently of sociocultural origin. However, visiting a gynaecological surgery as well as utilising contraception counselling correlates strongly with religious denomination and religious bond, as well as with having persons of trust and contact persons available for discussing sexual matters.

## 4. Discussion

The representative data from the current iteration of the Youth Sexuality Study shows that young people in Germany still have a variety of different sources and authorities at their disposal for obtaining information about sexual and reproductive health. In addition to the home, school still plays the most important role in terms of the institutionalised transfer of knowledge and skills. The significance of these authorities is confirmed in other, mostly international studies [20], whereby country-specific differences are observed [21].

The Internet is where young people obtain health information as well, which is also confirmed by other studies for the German population as a whole [22] and for the adolescent target group [23]. The sourcing offer is completed by professional sex and contraception counselling in certified counselling centres and gynaecological surgeries.

Sexuality education and contraception counselling in Germany are therefore organised intersectorally, and are based on many pillars. If one pillar is unavailable – for example the home – other authorities and offerers can compensate for this proportionately, and especially the school setting is particularly important here. Sexuality education is mandatory for all school types in Germany [24]. By attending school, all young people in Germany thus have access to fact-based health information in the field of sexuality and contraception. Furthermore, for Islamic girls and boys, and for girls and boys under strict religious influence and/or with low obtained or aspired levels of education, the school as the place for sexuality education provides important compensation for the fact that their parents are available more rarely as contact persons for sexual matters.

The fact that this combined effort from different sources and authorities has been highly successful for the evidence-based and skills-oriented health communication in Germany in the last few decades, is reflected in the high contraception competency of young people. When having intercourse for the first time, only 9% did not use contraceptives, during the most recent intercourse only 5% did not use contraceptives [8], while the uninterrupted drop in teenage pregnancies by more than two thirds since 2004 can be seen as indicator for the respective generations of young people’s high knowledge and safe behaviour in terms of contraception and sexuality [25].

Fact-based and skills-oriented concepts, media, and offerings for sexuality education, contraception and family planning, which institutions, associations and sponsors provide free of charge across Germany, are an essential element when it comes to boosting sexual and reproductive health among young people in Germany. Promoting behaviours and stimulating the motivation to make behavioural changes are, in addition to the transfer of knowledge, the central elements of these concepts and offerings. The offerings are aimed at both the target group of adolescents and their parents or of young adults, respectively, but also at disseminators in schools, medical surgeries and certified counselling centres.

This evidence-based health communication is all the more important because young people also use testimonials and recommendations from ‘health amateurs’ as relevant sources of information, which are found especially in the digital domain and on social media [26]. For example, influencers with large audiences make their personal experiences the centre of their messages, while scientific evidence is not represented in a well-balanced manner, and myths, even conspiracy theories are disseminated, especially in the context of contraceptives [26]. It is important here to empower young people to source and evaluate digital information in the field of sexual and reproductive health, and to further develop target group-specific digital offers that provide fact-based knowledge [27, 28]. Institutionalised sexuality education in schools, certified counselling centres and gynaecological surgeries function as a kind of antagonistic anthesis, and are of essential importance for the dissemination of evidence-based sexuality education to young people.

As part of the COVID-19 pandemic, the offering of sexuality education and contraception in Germany had to be stopped virtually completely [29]. The emergence of access barriers to preventative health services in this field as a result of the pandemic are also reported by international studies [30, 31]. The impact of this development on the sexual and reproductive health of young people in Germany will most likely be visible in the next representative iteration of the Youth Sexuality Study, which is currently being planned.

The overall aim is to maintain and to intensify the inter-sectoral commitment in the field of sexuality education and family planning. This is the only way to ensure the sexual and reproductive health of future generations, to reduce possible negative consequences of the COVID-19 pandemic, and to use fact-based and skills-oriented health information to counteract the experience-based messages in the digital domain.

Finally, it is worth mentioning that the findings at hand are based on self-reported information by young people in Germany, and that distortive answer tendencies based on social desirability can therefore not be ruled out. Indications pointing to a differential effectiveness of the individual sources, information paths and offerings relating to sexual and reproductive health, can also not be inferred based on the results at hand. Additional research efforts are called for here, which comparatively analyse the advantages and disadvantages of the sources for sexuality education in view of availability, utilisation and effects on the transfer of knowledge and skills, thus promoting good health.

## Key statement

- Sexuality education for adolescents in Germany is intersectorally organised.
- Institutionalised transfer of knowledge and practical skills in schools, and at certified counselling centres and in gynaecological counselling will guarantee the conveyance of evidence-based facts.
- Knowledge transfer and education through institutionalised settings is especially essential for those young people, who do not have a trusted contact person for sexual questions in their families.
- On the Internet, evidence-based messages and myths are also shared. Evidence-based and practice-oriented information are therefore an essential counterpart.

## Figures and Tables

**Table 1:** Percentage of girls and young women who have never visited a gynaecological surgery, according to religious denomination and strict religious bond (n=3,604, unweighted)^*^ Source: Youth Sexuality Study, 9th iteration (BZgA)

		Proportion (%)	χ^2^ (df)
Religious denomination	Lutheran	12	51,529(3)^**^
	Catholic	13	
	Islamic	30	
	Other/none	13	
Strict religious bond	Yes	26	60,985(1)^**^
	No	12	

*Group differences = two-sided χ^2^ test with

** p < 0.001

## Appendix

**Annex Table 1:** Questions and answer options of the used items of the 9th Iteration of the Youth Sexuality Study Source: Youth Sexuality Study, 9th iteration (BZgA)

Questions	Answer options	Database
What is the predominant source of your knowledge about sexuality, reproduction, contraception, etc.?	Multiple answers, list template	14- to 17-year-olds (n=3,556, unweighted)
	11: Discussions	
	12: Lectures	
	13: School lessons	
	14: Books	
	15: Magazines/newspapers	
	16: Youth journals	
	17: Complementary sexuality education leaflets	
	18: DVDs, videotapes	
	19: Television films	
	20: Radio	
	21: Computer programs, computer games	
	22: Internet	
	23: Own experience	
	98: Other (From where? Please describe briefly)	
Did you discuss sexuality education topics in class?	1: Yes	/*
	2: No	
Who were the most important persons for you for information about sexual matters?	Multiple answers, list template	14- to 17-year-olds (n=3,556, unweighted)
	11: Father	
	12: Mother	
	13: Teacher	
	14: Doctor	
	15: Brother	
	16: Sister	
	17: Friend or partner, respectively	
	18: The best friend	
	19: Other boys	
	20: Other girls	
	21: Youth group leader	
	22: Kindergarten staff	
	98: Other persons (Who? Please describe briefly)	
Does your family talk about sexuality and partnership?	1: Yes	14- to 17-year-olds (n=3,556, unweighted)
	2: No	
From which media would you prefer to obtain additional information about the topics mentioned by you?	Multiple answers, list template	14- to 25-year-olds (n=6,032, unweighted)
	11: Books	
	12: Magazines/newspapers	
	13: Public lectures	
	14: Helpline	
	15: Complementary sexuality education leaflets	
	16: Youth magazines	
	17: Sexuality education games, e.g. boardgames	
	18: DVDs	
	19: Television films	
	20: Radio	
	21: Comics	
	22: CDs	
	23: Computer programs, computer games	
	24: Internet	
	25: Public exhibitions	
	(Only 18- to 25-year-olds)	
	26: I do not want any additional information	
Have you found out something that is important to you about sexuality on the Internet yet?	1: No	14- to 25-year-olds (n=6,032, unweighted)
	2: Yes	
Assuming you want to source information about sexual matters you are interested in on the Internet, where do you look first?	Multiple answers, NO list template, but open answers	14- to 25-year-olds (n=6,032, unweighted)
	11: YouTube	
	12: Facebook	
	13: Instagram	
	14: Twitter	
	77: Wikipedia	
	88: Simply by ‘Googling’ (search engines)	
	97: Other, namely: (Please describe briefly)	
	98: I do not use digital media to look for information	
Where did you find out something about sexuality that was important to you?	Multiple answers, list template	14- to 25-year-olds, who indicate that they’ve found out something important about sexuality on the Internet (N=4,112, unweighted)
	11: Wikipedia	
	12: Sexuality education or counselling sites	
	13: Forums on which experts answer questions	
	14: Forums on which other forum visitors answer questions	
	15: Chats with others	
	16: Sex films I have watched	
	17: Influencers	
	98: Other, namely: (Please describe briefly)	
From which persons would you prefer to get additional information about the topics mentioned by you?	Multiple answers, list template	14- to 17-year-olds (n=3,556, unweighted)
	11: Father	
	12: Mother	
	13: Teacher	
	14: Doctor	
	15: Experts in a certified counselling centre	
	16: Brother	
	17: Sister	
	18: Friend or partner, respectively	
	19: Other boys	
	20: Other girls	
	21: Other persons	
	22: I do not want additional information	
How old were you when you first visited a gynaecologist?	Open answer	14- to 25-year-old girls/ young women (n=3,604, unweighted)
What was the reason for this first visit to the gynaecologist’s?	Multiple answers, list template	14- to 25-year-old young women, who visited a gynaecologist (n=2,797, unweighted)
	1: (Problems with) menstruation, menstrual bleeding	
	2: Contraception	
	3: Fear of being pregnant	
	4: Unclear abdominal pain	
	5: HPV vaccination (vaccination against human papillomavirus)	
	8: Something else (What? Please describe briefly)	

^*^ Database cannot be specified because the analysis refers to the trend of the past nine iterations
